# Impulsive control for synchronizing delayed discrete complex networks with switching topology

**DOI:** 10.1007/s00521-013-1470-3

**Published:** 2013-08-24

**Authors:** Chaojie Li, David Y. Gao, Chao Liu, Guo Chen

**Affiliations:** 1 School of Science, Information Technology and Engineering, University of Ballarat, Mt Helen, VIC 3350 Australia; 2 School of Electrical and Information Engineering, Chongqing University of Science and Technology, Chongqing, 401331 People’s Republic of China; 3 School of Electrical and Information Engineering, The University of Sydney, Sydney, 2006 Australia

**Keywords:** Impulsive synchronization, Complex networks, Switching topology, Lyapunov-Ruzimiki method

## Abstract

In this paper, global exponential synchronization of a class of discrete delayed complex networks with switching topology has been investigated by using Lyapunov-Ruzimiki method. The impulsive scheme is designed to work at the time instant of switching occurrence. A time-varying delay-dependent criterion for impulsive synchronization is given to ensure the delayed discrete complex networks switching topology tending to a synchronous state. Furthermore, a numerical simulation is given to illustrate the effectiveness of main results

## Introduction

It has long been understood that many physical, social, biological, and technological networks are modeled by a graph with non-trivial topological features. In this model, every node is an individual element of the whole system with certain pattern of connections, in which connections between each pair of nodes are neither entirely regular nor entirely random [1–3]. These features do not occur in the mathematical models of networks that have been studied in the past, such as lattices or random graphs, but they do truly exist in nature. At present, derivatives of network science have been successfully applied to the analysis of metabolic and genetic regulatory networks, the design of robust and scalable communication networks both wired and wireless, the development of vaccination strategies for the control of disease, and a broad range of other practical issues.

Secure communication [4, 5], parallel image processing [6], and chemical reaction implemented by coupled chaotic systems have been an active research field during the last two decades. Synchronization issues are fundamentally important for any dynamical networks, and there is no exception for complex networks. As a consequence, theory and methods for synchronization of different families of complex networks have been extensively studied by many researchers (such as [7–15], and references therein). Network synchronization has been discussed in terms of spectral and statistical properties by authors [16]. The results on complete synchronization, phase synchronization, space lag synchronization, and cluster reflect the ideas of [17–19]. The improvement on different regimes of synchronization of discrete complex networks is abstracted from papers authored by [9, 20]. Some general cases of synchronization of complex networks with switching topology can be found in the literatures of [15, 21]. From another angle, several approaches to synchronize a complex network have been proposed. Adaptive synchronization, impulsive synchronization scheme, and pining control synchronization have been considered by authors in [10–12, 22, 23–27].  In addition, systems with delays and multiplicative noises have been studied by considering H-infinity method in [28] and [29].

However, all previous studies have limitations on synchronizing a state delayed discrete complex network. As known, connections between each pair of nodes in a complex network always change. The mutable topology can pose a significant threat on the global dynamical property of the whole networks. Indeed, the fact has been ignored by most of pioneering work. Additionally, the large time-varying delays may exist in switching topology which means data communication may occur in different sub-networks. It is much more complicated than previous studies. If the mutable topology and a large time-varying delay occur simultaneously in the discrete complex networks, it would be difficult to employ previous synchronization control schemes. Therefore, it is necessary to investigate new synchronization method.

Impulsive control has been successfully used to stabilize and synchronize dynamical systems, for examples, [30–37]. And impulsive control technique could be an efficient method when a discrete change behavior is needed. The adjustment interest rate could agree with that. In this paper, we proposed an impulsive synchronization scheme for a state delayed discrete complex network with switching topology. For this control scheme, we consider that the impulsive control signal is designed to be input into all of nodes. Meanwhile, every time instant of impulsive effects occur precisely at the time instant of switching happening. In other words, when the complex networks switch its state at every instant of time, there is no delay between the controller functioning.

The paper is organized as follows. Section 2 presents some mathematical preliminaries needed in this work, and a generalized mathematical model for delayed discrete complex networks with switching topology. The main theorem for global synchronization of this type of discrete complex networks is then given in Sect. 3. In Sect. 4, a small-world network with 3 sub-networks involving 30 nodes is constructed to illustrate the effectiveness of our result. Section 5 concludes the paper.

## Preliminary

First, we need to introduce some notations and definitions for the sake of exploring our main results. Let $\|\bullet\|$ denote the Euclidean norm; ${\mathbb{R}^n}$ denotes the *n* dimensional Euclidean space, the set of natural numbers ${\mathbb{N}=\{0,1,2,\ldots\}}$, and, for certain positive integer *τ*, we let ${\mathbb{Z}_{-\tau}=\{-\tau,-\tau+1,\ldots,0\}}$. The family of *N* linearly coupled discrete complex networks, consisting of time delay with respect to its system state and the switched topology, can be described by

1 $$\begin{aligned} x_i(k+1)&=A x_i(k)+ B f(x_i(k))+ D f(x_i(k-\tau(k)))+I(k) \\ &+\sum\limits_{j=1}^Nc_{ij,\sigma(k)}\Upgamma x_j(k-\tau(k)), \quad i=1,2,\ldots ,N,k\in {\mathbb{N}} \end{aligned}$$

2 $$x_{ik_0}=\phi(\theta),\quad \theta \in {\mathbb{Z}}_{-\tau},$$

where ${x_i(k)=(x_{i,1},x_{i,2},\ldots ,x_{i,n})\; \in \mathbb{R}^n}$ represents the state vector of the *i*-th node at every instant of time *k* and *n* denotes the number of nodes affiliated to each sub-network. ${{\bf A} \in \mathbb{R}^{n\times n},\;{\bf B} \in \mathbb{R}^{n\times n}}$ and ${{\bf D} \in \mathbb{R}^{n\times n}}$ are known real matrices. ${\bf f(x_i(k))}=({\bf f_1(x_{i,1}(k))},{\bf f_2(x_{i,2}(k))},\;\ldots,{\bf f_n(x_{i,n}(k))})^{T}$ and ${{\bf f}(\bullet):\mathbb{R}^n \longrightarrow \mathbb{R}^n}$ is a smooth nonlinear vector-valued functions. ${\bf I(k)}=(I_1(k),I_2(k),\;\ldots,I_n(k))^{T}$ is a n-dimensional vector from external input. **S** is a finite index set of *r* elements: ${\bf S}=\{s_1,s_2,\ldots ,s_r\}$. Let the switching function be denoted by ${\sigma(k):\mathbb{N} \longrightarrow {\bf S}}$, which is the switching signal from sudden changing of system dynamic without jumps in the state **x** at any switching instant. Specifically, we consider that it is a piecewise constant function and continuous from the right, indicating certain active sub-system regime, at every instant of time *k* the index $\sigma(k)=s_k \in {\bf S}$; meanwhile, let the switching instants of σ be denoted by $k_{m,x}\;(m=1,2,\ldots)$ and let *k*_0,*x*_: = 0. ${C_{s_k}=(c_{ij,s_k})\in \mathbb{Z}^{N\times N}}$ represents the outer coupling configuration symmetric matrix defined as follows: For each active sub-system regime *s*_*k*_, if there is a connection from node *j* to node *i* (*j* ≠ *i*), then $c_{ij,s_k}=c_{ji,s_k}>0$; otherwise $c_{ij,s_k}=c_{ji,s_k}=0$. Assume that

3 $$c_{ii,s_k}=-\sum\limits_{j=1,j\neq i}^{N}c_{ij,s_k}=-\sum\limits_{j=1,j\neq i}^{N}c_{ji,s_k}, i\in N, s_k\in {\bf S}.$$

The notation ${\Upgamma\in \mathbb{R}^{n\times n}}$ represents the diagonal inner coupling matrix between two connected nodes. τ(*k*) is a time-varying delay with respect to each instant of time *k* and satisfies ${\tau(k)\in \mathbb{Z}_{-\tau}}$. ${\phi(\bullet):\mathbb{Z}_{-\tau}\longrightarrow \mathbb{R}^{n\times N}}$ is smooth everywhere except at a finite number of points. The norm of ϕ(•) is defined by ${\|\phi(\theta)\|_{\tau}=\sup_{\theta \in \mathbb{Z}_{-\tau}}\{\|\phi(\theta)\|\}}$.

In order to design an impulsive control scheme to synchronize system (1), we consider the evolutionary state is abruptly jumping at every impulsive instant of time *k*_*u*_ from its open-loop state, which can be formulated by

4 $$\Updelta x_i(k_{m,u})=J_u x_{i}^{*}(k_{m,u}),\quad m=1,2,\ldots {\mathbb{N}}$$

where *x*_i_^*^(*k*_*m*,*u*_) stands for the primal state at time instant *k*_*m*,*u*_ without impulsive jump. As usual, every impulsive instant of time *k*_*l*,*u*_ satisfies $0=k_{0,u}<k_{1,u}<k_{2,u}<\cdots<k_{m,u}<k_{m+1,u}<\ldots$ and ${\lim\nolimits_{m\rightarrow \infty}k_{m,u}=\infty;\;J_u:\mathbb{R}^{n}\rightarrow \mathbb{R}^{n}(m=1,2,\ldots)}$ represents the impulsive jump strength. Therefore, at every impulsive instant of time *k*_*m*,*u*_, the coupled states *x*_*i*_(*k*) − *x*_*j*_(*k*) between connected node *i* and *j* can be described by

5 $$\begin{aligned} x_i(k_{m,u})-x_j(k_{m,u})&=x_{i}^{*}(k_{m,u})-x^{*}_{j}(k_{m,u}) \\ & \quad+J_u[x_{i}^{*}(k_{m,u})-x^{*}_{j}(k_{m,u})]. \end{aligned}$$

Intuitively, a family of impulsive controller can be designed as

6 $$U_i(k,x_i(k))=\sum\limits_{u=1}^{\infty}\delta(k-k_{m,u})J_u(x_{i}^{*}(k_{m,u})), \quad m=1,2,\ldots {\mathbb{N}},$$

where *U*_*i*_(*k*, *x*_*i*_(*k*)) represents a class of impulsive controller at each instant of time $k_{m,u};\;\delta(\bullet)$ denotes the Dirac discrete-time function. Correspondingly, by virtue of impulsive controller, the closed-loop discrete complex networks can be derived by the following form:

7 $$\begin{aligned} x_i(k+1)&=A x_i(k)+ B f(x_i(k))+ D f(x_i(k-\tau(k)))+I(k) \\ & \quad +\sum\limits_{j=1}^Nc_{ij,\sigma(k)}\Upgamma x_j(k-\tau(k))+U_i(k+1,x_i^*(k+1)), \\ &\quad i=1,2,\ldots ,N. \end{aligned}$$

8 $$\begin{aligned} U_i(k+1,x_i^*(k+1))&=\sum\limits_{u=1}^{\infty}\delta(k+1-k_{m,u})J_u[A x_i(k) \\ & \quad +B f(x_i(k))+ D f(x_i(k-\tau(k))) +I(k) \\ & +\sum\limits_{j=1}^Nc_{ij,\sigma(k)}\Upgamma x_j(k-\tau(k))], \quad m=1,2,\ldots ,{\mathbb{N}}. \end{aligned}$$

### **Assumption 1**

For each nonlinear function ${\bf f_i(\bullet)}\;(i=1,2,\ldots ,n)$, suppose that it is globally Lipschitz continues function and satisfies

9 $$\|f_i(x_1)-f_i(x_2)\| \leq \hat l_i\|x_1-x_2\|,\quad i=1,2,\ldots ,n, \forall x_1,x_2 \in {\mathbb{R}},$$

where $\hat l_i$ is certain positive constant.

### **Definition 1**

The system of the impulsive controlled discrete complex networks (7) is said to be globally exponentially synchronized, if for any initial condition ${\phi (\bullet):\mathbb{Z}_{-\tau}\rightarrow\mathbb{R}^{n\times N}}$, and there exist two positive constants λ and *M*_0_ ≥ 1 such that

10 $$\|x_i(k)-x_j(k)\|\leq M_0 \text{e}^{-\lambda(k-k_0)},\quad 1\leq i \leq j\leq N$$

holds for all *k* > *k*_0_.

### **Lemma**

[18] *Let* ${{\bf W}=(w_{ij})_{N\times N},\;{\bf P} \in \mathbb{R}^{n\times n},\;{\bf x}=(x_1,x_2,\;\ldots,x_N)^{T}}$ *and* ${\bf y}=(y_1,y_2,\ldots ,y_N)^{T}$ *with* ${x_k,\;y_k \in \mathbb{R}^{N}\;(k=1,2,\ldots ,N)}$. If **W** = **W** ^*T*^ and each row sum of **W** is zero, then

11 $${\bf x}^{{\bf T}}({\bf W}\otimes {\bf P}){\bf y}=-\sum\limits_{i=1}^{N-1}\sum\limits_{j=i+1}^{N}w_{ij}(x_i-x_j)^{T}P(y_i-y_j).$$

## Main results

When the impulsive controller can be functioning simultaneously at the state of discrete complex networks’ switching signal, the equivalent impulsive controlled system is rewritten by using the matrix Kronecker product (see [38])

12 $$\begin{aligned} x(k+1)&=(I_N\otimes A)x(k)+(I_N\otimes B)F(x(k)) \\ & \quad +(I_N\otimes D)F(x(k-\tau)) +I(k)+(C_{\sigma (k)}\otimes \Upgamma)x(k-\tau), \\ & \quad k\neq k_{m,u} \end{aligned}$$

13 $$x(k_{m,u})=[I_N\otimes (I_N+J_u(k_{m,u}))]x(k_{m,u}-1),$$

for any $${k,\;m\in \mathbb{N}}$$.

### **Theorem 1**

Under **Assumption 1**., the impulsive controlled complex network (12) is exponentially synchronized if there exist certain positive integer *m*_τ_, positive scalars $\varepsilon_{\sigma (k)},\;p_{\sigma (k)},\;q_{\sigma (k)}$, and positive-definite matrices ${P_{\sigma (k)}\in \mathbb{R}^{n\times n},\;Q_{l,\sigma (k)}\in \mathbb{R}^{n\times n}\;(l=1,2,\ldots 6)}$ such that

- Given *μ* ≥ 1 and $P_{\sigma (k_{m,x})}\le \mu P_{\sigma (k_{m+1,x})}$, for any $k\in [k_{m,x},k_{m+1,x}-1]$ in corresponding sub-state σ(*k*_*m*,*x*_),
  14 $$p_{\sigma(k_{m,x})}-\left[\frac{\lambda_{\rm max}(\Uppi_{\sigma(k_{m,x})})} {\lambda_{\rm min}\left(P_{\sigma(k_{m,x})}^{-1}\right)}+ \mu q_{\sigma(k_{m,x})} \frac{\lambda_{\rm max}(\Upomega_{\sigma(k_{m,x})})}{\lambda_{\rm min} \left(P_{\sigma(k_{m-m_{\tau},x})}^{-1}\right)}\right]\geq0,$$
  where
  $$\begin{aligned} \Uppi_{\sigma(k_{m,x})}&=A^{T}P_{\sigma(k_{m,x})}A+L^{T}B^{T}P_{\sigma(k_{m,x})}BL \\ & \quad +L^{T}B^{T}P_{\sigma(k_{m,x})}^{T}Q_{1,\sigma(k_{m,x})}P_{\sigma(k_{m,x})}BL \\ & \quad +A^{T}Q_{1,\sigma(k_{m,x})}A +A^{T}Q_{2,\sigma(k_{m,x})}^{-1}A \\ & \quad -NC_{\sigma(k_{m,x})}A^{T}Q_{3,\sigma(k_{m,x})}^{-1}AC_{\sigma(k_{m,x})} \\ & \quad -L^{T}NC_{\sigma(k_{m,x})}B^{T}Q_{5,\sigma(k_{m,x})}BC_{\sigma(k_{m,x})}L \\ & \quad +L^{T}B^{T}Q_{4,\sigma(k_{m,x})}BL \\ \Upomega_{\sigma(k_{m,x})}&=L^{T}D^{T}P_{\sigma(k_{m,x})} DL-NC_{\sigma(k_{m,x})}^{2}\Upgamma^{T}P_{\sigma(k_{m,x})}\Upgamma \\ & \quad +L^{T}D^{T}P_{\sigma(k_{m,x})}^{T}Q_{2,\sigma(k_{m,x})}P_{\sigma(k_{m,x})}DL \\ & \quad -\Upgamma^{T}P_{\sigma(k_{m,x})}^{T}Q_{3,\sigma(k_{m,x})}P_{\sigma(k_{m,x})}\Upgamma \\ & \quad +L^{T}D^{T}P_{\sigma(k_{m,x})}^{T}Q_{4,\sigma(k_{m,x})}P_{\sigma(k_{m,x})}DL \\ & \quad -\Upgamma^{T}P_{\sigma(k_{m,x})}^{T}Q_{5,\sigma(k_{m,x})}P_{\sigma(k_{m,x})}\Upgamma \\ & \quad -L^{T}NC_{\sigma(k_{m,x})}D^{T}Q_{6,\sigma(k_{m,x})}^{-1}DC_{\sigma(k_{m,x})}L \\ & \quad -\Upgamma^{T}P_{\sigma(k_{m,x})}^{T}Q_{6,\sigma(k_{m,x})}P_{\sigma(k_{m,x})}\Upgamma. \\ \end{aligned}$$
- $$\mu\lambda_{\rm max}^{2}(1+J_u(k_{m,x}))<\text{e}^{\varepsilon_{\sigma(k_{m,x})} (k_{m+1,x}-k_{m,x})}$$.
- $$q_{\sigma(k_{m,x})}\geq \text{e}^{\varepsilon_{\sigma(k_{m,x})}(k_{m+1,x}-k_{m,x}+1)+ \sum\nolimits_{i=0}^{m_{\tau}-1}\varepsilon_{k_{m-i,x}}(k_{m+1-i,x}-k_{m-i,x})}$$, where $$m_{\tau}=\lceil\frac{\tau}{\inf\{k_{m,x}-k_{m-1,x}\}}\rceil$$.

### *Proof*

Consider the following Lyapunov function:

15 $$V(k)=x^{T}(k)(W\otimes P_{\sigma(k)})x(k),$$

for any $k\in[k_{m,x},k_{m+1,x}-1],\;\quad m=1,2,\ldots$ where

$$W=\left[\begin{array}{cccc} N-1 & -1 & \ldots & -1 \\ -1 & N-1 & \ldots & -1 \\ \ldots & \ldots & \ldots & \ldots \\ -1 & -1 & \ldots & N-1\end{array}\right].$$

Let $\varphi(\|\phi_{i}(\theta)-\phi_{j}(\theta)\|^{2}) =\lambda_{\rm max}(P_{\sigma(\theta)})\|\phi_{i}(\theta)-\phi_{j}(\theta)\|^{2}$, thus for the case ${k\in \mathbb{Z}_{\tau}}$,

16 $$\begin{aligned} V(\theta)&=x^{T}(\theta)(W\otimes P_{\sigma(0)})x(\theta) \\ &=\sum\limits_{i=1}^{N-1}\sum\limits_{j=i+1}^{N}(x_i(\theta)-x_j(\theta))^{T}P_{\sigma(0)} (x_i(\theta)-x_j(\theta)) \\ &\leq \varphi(\|\phi_i(\theta)-\phi_j(\theta)\|_{\tau}^{2}) \\ &=\varphi(\|\phi(\theta)\|_{\tau}^{2}). \end{aligned}$$

Choose *M* ≥ 1, such that

17 $$\begin{aligned} \varphi(\|\phi(\theta)\|_{\tau}^{2})&\leq M\varphi(\|\phi(\theta)\|_{\tau}^{2}) \text{e}^{-\lambda(k_{1,x}-k_{0,x})}\text{e}^{-\varepsilon_{\sigma(k_{0,x})}(k_{1,x}-k_{0,x})} \\ &<M\varphi(\|\phi(\theta)\|_{\tau}^{2})\text{e}^{-\lambda(k_{1,x}-k_{0,x})} \\ &<q_{\sigma(k_{0,x})}\varphi(\|\phi(\theta)\|_{\tau}^{2}). \end{aligned}$$

By claiming that

18 $$V(k)\leq M\varphi(\|\phi(\theta)\|_{\tau}^{2})\text{e}^{-\lambda(k_{m,x}-k_{0,x})}, k\in [k_{m-1,x},k_{m,x}-1], m\in N.$$

We first need to show that

19 $$V(k)\leq M\varphi(\|\phi(\theta)\|_{\tau}^{2})\text{e}^{-\lambda(k_{1,x}-k_{0,x})},k\in [k_{0,x},k_{1,x}-1].$$

Obviously, for $$k\in [k_{0,x}-\tau,k_{0,x}-1]$$,

20 $$\begin{aligned} V(k)&\leq \varphi(\|\phi(\theta)\|_{\tau}^{2}) \\ &\leq M\varphi(\|\phi(\theta)\|_{\tau}^{2}) \text{e}^{-\lambda(k_{1,x}-k_{0,x})}\text{e}^{-\varepsilon_{\sigma(k_{0,x})}(k_{1,x}-k_{0,x})}. \end{aligned}$$

If (19) is not true, then there exists certain instant of time $\bar{k}\in [k_{0,x},k_{1,x}-1]$ satisfying

21 $$\bar{k}=\text{min} \left \{k\in[k_{0,x},k_{1,x}-1]:V(k) >M\varphi(\|\phi(\theta)\|_{\tau}^{2})\text{e}^{-\lambda(k_{1,x}-k_{0,x})}\right\}$$

such that

22 $$\begin{aligned} V(\bar{k})&>M\varphi(\|\phi(\theta)\|_{\tau}^{2})\text{e}^{-\lambda(k_{1,x}-k_{0,x})} \\ &>M\varphi(\|\phi(\theta)\|_{\tau}^{2})\text{e}^{-\lambda(k_{1,x}-k_{0,x})} \text{e}^{-\varepsilon_{\sigma(k_{0,x})}(k_{1,x}-k_{0,x})} \\ &>\varphi(\|\phi(\theta)\|_{\tau}^{2}). \\ \end{aligned}$$

and

23 $$V(k)\leq M\varphi(\|\phi(\theta)\|_{\tau}^{2})\text{e}^{-\lambda(k_{1,x}-k_{0,x})}, k\in [k_{0,x}-\tau,\bar{k}-1]$$

Considering that

24 $$k^{\ast}= \text{max} \left \{k\in[k_{0,x},\bar{k}]:V(k) \leq M\varphi(\|\phi(\theta)\|_{\tau}^{2})\text{e}^{-\lambda(k_{1,x}-k_{0,x})} \text{e}^{-\varepsilon_{\sigma(k_{0,x})}(k_{1,x}-k_{0,x})}\right\},$$

from which we have

25 $$V(k)> M\varphi(\|\phi(\theta)\|_{\tau}^{2})\text{e}^{-\lambda(k_{1,x}-k_{0,x})} \text{e}^{-\varepsilon_{\sigma(k_{0,x})}(k_{1,x}-k_{0,x})}, k\in [k^{\ast}+1,\bar{k}-1].$$

Therefore,

26 $$V(k^{\ast})\leq V(k)\leq V(\bar{k}),k\in [k^{\ast},\bar{k}].$$

And for any $k\in [k^{\ast},\bar{k}-1]$,

27 $$\begin{aligned} V(k+s)&\leq M\varphi(\|\phi(\theta)\|_{\tau}^{2})\text{e}^{-\lambda(k_{1,x}-k_{0,x})} \\ &\leq \text{e}^{\varepsilon_{\sigma(k_{0,x})}(k_{1,x}-k_{0,x})}V(k^{\ast}) \\ &\leq q_{\sigma(k_{0,x})}V(k). \end{aligned}$$

Consider the increment of *V*(*k*) along the solution of discrete complex networks (12) in the interval $k\in [k^{\ast},\bar{k}-1]$, one observes

28 $$\begin{aligned}   \Delta V(k) &  = V(k + 1) - V(k) \\     & \; = [(I_{N}  \otimes A)x(k) + (I_{N}  \otimes B)F(x(k)) \\    \quad  + (I_{N}  \otimes D)F(x(k - \tau )) + I(k) \\    \quad  + (C_{{\sigma (k)}}  \otimes \Gamma )x(k - \tau){]}^{T} (W \otimes P_{{\sigma (k + 1)}} )[(I_{N}  \otimes A)x(k) \\    \quad  + (I_{N}  \otimes B)F(x(k)) + (I_{N}  \otimes D)F(x(k - \tau )) \\    \quad  + I(k) + (C_{{\sigma (k)}}  \otimes \Gamma )x(k - \tau )] - x^{T} (k)P_{{\sigma (k)}} x(k) \\     & \; = [(I_{N}  \otimes A)x(k) + (I_{N}  \otimes B)F(x(k)) \\    \quad  + (I_{N}  \otimes D)F(x(k - \tau )) + I(k) \\    \quad  + (C_{{\sigma (k)}}  \otimes \Gamma )x(k - \tau ){]}^{T} (W \otimes P_{{\sigma (k_{{0,x}} )}} )[(I_{N}  \otimes A)x(k) \\    \quad  + (I_{N}  \otimes B)F(x(k)) + (I_{N}  \otimes D)F(x(k - \tau )) \\    \quad  + I(k) + (C_{{\sigma (k)}}  \otimes \Gamma )x(k - \tau )] - x^{T} (k)P_{{\sigma (k_{{0,x}} )}} x(k) \\     & \; = x^{T} (k)(I_{N}  \otimes A^{T} )(W \otimes P_{{\sigma (k_{{0,x}} )}} )(I_{N}  \otimes A)x(k) \\    \quad  + F^{T} (x(k))(I_{N}  \otimes B^{T} )(W \otimes P_{{\sigma (k_{{0,x}} )}} )(I_{N}  \otimes B)F(x(k)) \\    \quad  + F^{T} (x(k - \tau ))(I_{N}  \otimes D^{T} )(W \otimes P_{{\sigma (k_{{0,x}} )}} )(I_{N}  \otimes D)F(x(k - \tau )) \\    \quad  + I^{T} (k)(W \otimes P_{{\sigma (k_{{0,x}} )}} )I(k) \\    \quad  + x^{T} (k - \tau )(C_{{\sigma (k_{{0,x}} )}}^{T}  \otimes \Gamma ^{T} )(W \otimes P_{{\sigma (k_{{0,x}} )}} )(C_{{\sigma (k_{{0,x}} )}}  \otimes \Gamma )x(k - \tau ) \\    \quad  + 2x^{T} (k)(I_{N}  \otimes A^{T} )(W \otimes P_{{\sigma (k_{{0,x}} )}} )(I_{N}  \otimes B)F(x(k)) \\    \quad  + 2x^{T} (k)(I_{N}  \otimes A^{T} )(W \otimes P_{{\sigma (k_{{0,x}} )}} )(I_{N}  \otimes D)F(x(k - \tau )) \\    \quad  + 2x^{T} (k)(I_{N}  \otimes A^{T} )(W \otimes P_{{\sigma (k_{{0,x}} )}} )I(k) \\    \quad  + 2x^{T} (k)(I_{N}  \otimes A^{T} )(W \otimes P_{{\sigma (k_{{0,x}} )}} )(C_{{\sigma (k_{{0,x}} )}}  \otimes \Gamma )x(k - \tau ) \\    \quad  + 2F^{T} (x(k))(I_{N}  \otimes B^{T} )(W \otimes P_{{\sigma (k_{{0,x}} )}} )(I_{N}  \otimes D)F(x(k - \tau )) \\    \quad  + 2F^{T} (x(k))(I_{N}  \otimes B^{T} )(W \otimes P_{{\sigma (k_{{0,x}} )}} )I(k) \\    \quad  + 2F^{T} (x(k))(I_{N}  \otimes B^{T} )(W \otimes P_{{\sigma (k_{{0,x}} )}} )(C_{{\sigma (k_{{0,x}} )}}  \otimes \Gamma )x(k - \tau ) \\    \quad  + 2F^{T} (x(k))(I_{N}  \otimes B^{T} )(W \otimes P_{{\sigma (k_{{0,x}} )}} )(I_{N}  \otimes D)F(x(k - \tau )) \\    \quad  + 2F^{T} (x(k))(I_{N}  \otimes B^{T} )(W \otimes P_{{\sigma (k_{{0,x}} )}} )I(k) \\    \quad  + 2F^{T} (x(k))(I_{N}  \otimes B^{T} )(W \otimes P_{{\sigma (k_{{0,x}} )}} )(C_{{\sigma (k_{{0,x}} )}}  \otimes \Gamma )x(k - \tau ) \\    \quad  + 2F^{T} (x(k - \tau ))(I_{N}  \otimes D^{T} )(W \otimes P_{{\sigma (k_{{0,x}} )}} )I(k) \\    \quad  + 2F^{T} (x(k - \tau ))(I_{N}  \otimes D^{T} )(W \otimes P_{{\sigma (k_{{0,x}} )}} )(C_{{\sigma (k_{{0,x}} )}}  \otimes \Gamma )x(k - \tau ) \\    \quad  + 2I^{T} (k)(W \otimes P_{{\sigma (k_{{0,x}} )}} )(C_{{\sigma (k_{{0,x}} )}}  \otimes \Gamma )x(k - \tau ) \\    \quad  - x^{T} (k)P_{{\sigma (k_{{0,x}} )}} x(k) \\  \end{aligned}$$

Note that

29 $$(W\otimes P_{\sigma(k)})I(k)=0 \quad and \quad I^{T}(k)(W\otimes P_{\sigma(k)})=0.$$

For the simplicity of calculation, we define

30 $$C_{\sigma (k_{0,x})}W C_{\sigma (k_{0,x})}=N C_{\sigma(k_{0,x})}^{2}.$$

31 $$WC_{\sigma (k_{0,x})}=NC_{\sigma (k_{0,x})}.$$

32 $$x_{ij}(k)=x_i(k)-x_j(k).$$

33 $$x_{ij}(k-\tau)=x_i(k-\tau)-x_j(k-\tau).$$

34 $$F_{ij}(x(k))=F_i(x(k))-F_j(x(k)).$$

35 $$F_{ij}(x(k-\tau))=F_i(x(k-\tau))-F_j(x(k-\tau)).$$

According to **Lemma 1**, we can obtain that

36 $$\begin{aligned}   \Delta V(k) &  = V(k + 1) - V(k) \\     & \; = \sum\limits_{{i = 1}}^{{N - 1}} {\sum\limits_{{j = i + 1}}^{N} {[x_{{ij}}^{T} (k)A^{T} P_{{\sigma (k_{{0,x}} )}} Ax_{{ij}} (k)} }  \\    \quad  + x_{{ij}}^{T} (k)L^{T} B^{T} P_{{\sigma (k_{{0,x}} )}} BLx_{{ij}} (k) \\    \quad  + x_{{ij}}^{T} (k - \tau )L^{T} D^{T} P_{{\sigma (k_{{0,x}} )}} DLx_{{ij}} (k - \tau ) \\    \quad  + x_{{ij}}^{T} (k - \tau )NC_{{\sigma (k_{{0,x}} )}}^{2} \Gamma ^{T} P_{{\sigma (k_{{0,x}} )}} \Gamma x_{{ij}} (k - \tau ) \\    \quad  + x_{{ij}}^{T} (k)A^{T} Q_{{1,\sigma (k_{{0,x}} )}}^{{ - 1}} Ax_{{ij}} (k) \\    \quad  + x_{{ij}}^{T} (k)L^{T} B^{T} P_{{\sigma (k_{{0,x}} )}}^{T} Q_{{1,\sigma (k_{{0,x}} )}} P_{{\sigma (k_{{0,x}} )}} BLx_{{ij}} (k) \\    \quad  + x_{{ij}}^{T} (k)A^{T} Q_{{2,\sigma (k_{{0,x}} )}}^{{ - 1}} Ax_{{ij}} (k) \\    \quad  + x_{{ij}}^{T} (k - \tau )L^{T} D^{T} P_{{\sigma (k_{{0,x}} )}}^{T} Q_{{2,\sigma (k_{{0,x}} )}} P_{{\sigma (k_{{0,x}} )}} DLx_{{ij}} (k - \tau ) \\    \quad  - x_{{ij}}^{T} (k)NC_{{\sigma (k_{{0,x}} )}} A^{T} Q_{{3,\sigma (k_{{0,x}} )}}^{{ - 1}} AC_{{\sigma (k_{{0,x}} )}} x_{{ij}} (k) \\    \quad  - x_{{ij}}^{T} (k - \tau )\Gamma ^{T} P_{{\sigma (k_{{0,x}} )}}^{T} Q_{{3,\sigma (k_{{0,x}} )}} P_{{\sigma (k_{{0,x}} )}} \Gamma x_{{ij}} (k - \tau ) \\    \quad  + x_{{ij}}^{T} (k)L^{T} B^{T} Q_{{4,\sigma (k_{{0,x}} )}}^{{ - 1}} BLx_{{ij}} (k) \\    \quad  + x_{{ij}}^{T} (k - \tau )L^{T} D^{T} P_{{\sigma (k_{{0,x}} )}}^{T} Q_{{4,\sigma (k_{{0,x}} )}} P_{{\sigma (k_{{0,x}} )}} DLx_{{ij}} (k - \tau ) \\    \quad  - x_{{ij}}^{T} (k)L^{T} NC_{{\sigma (k_{{0,x}} )}} B^{T} Q_{{5,\sigma (k_{{0,x}} )}}^{{ - 1}} BC_{{\sigma (k_{{0,x}} )}} Lx_{{ij}} (k) \\    \quad  - x_{{ij}}^{T} (k - \tau )\Gamma ^{T} P_{{\sigma (k_{{0,x}} )}}^{T} Q_{{5,\sigma (k_{{0,x}} )}} P_{{\sigma (k_{{0,x}} )}} \Gamma x_{{ij}} (k - \tau ) \\    \quad  - x_{{ij}}^{T} (k - \tau )L^{T} NC_{{\sigma (k_{{0,x}} )}} D^{T} Q_{{6,\sigma (k_{{0,x}} )}}^{{ - 1}} DC_{{\sigma (k_{{0,x}} )}} Lx_{{ij}} (k - \tau ) \\    \quad  - x_{{ij}}^{T} (k - \tau )\Gamma ^{T} P_{{\sigma (k_{{0,x}} )}}^{T} Q_{{6,\sigma (k_{{0,x}} )}} P_{{\sigma (k_{{0,x}} )}} \Gamma x_{{ij}} (k - \tau ) \\    \quad  - x_{{ij}}^{T} (k)P_{{\sigma (k_{{0,x}} )}} x_{{ij}} (k)] \\     & \; = \sum\limits_{{i = 1}}^{{N - 1}} {\sum\limits_{{j = i + 1}}^{N} {\{ x_{{ij}}^{T} (} } k)[A^{T} P_{{\sigma (k_{{0,x}} )}} A + A^{T} Q_{{1,\sigma (k_{{0,x}} )}} A \\    \quad  + L^{T} B^{T} P_{{\sigma (k_{{0,x}} )}} BL + A^{T} Q_{{2,\sigma (k_{{m,x}} )}}^{{ - 1}} A \\    \quad  + L^{T} B^{T} P_{{\sigma (k_{{m,x}} )}}^{T} Q_{{1,\sigma (k_{{m,x}} )}} P_{{\sigma (k_{{m,x}} )}} BL \\    \quad  - NC_{{ij,\sigma (k_{{0,x}} )}} A^{T} Q_{{3,\sigma (k_{{0,x}} )}}^{{ - 1}} AC_{{ij,\sigma (k_{{0,x}} )}}  \\    \quad  + L^{T} B^{T} Q_{{4,\sigma (k_{{0,x}} )}} BL \\    \quad  - L^{T} NC_{{\sigma (k_{{0,x}} )}} B^{T} Q_{{5,\sigma (k_{{0,x}} )}} BC_{{\sigma (k_{{0,x}} )}} L - P_{{\sigma (k_{{0,x}} )}} ]x_{{ij}} (k) \\    \quad  + x_{{ij}}^{T} (k - \tau )[L^{T} D^{T} P_{{\sigma (k_{{0,x}} )}} DL - NC_{{\sigma (k_{{0,x}} )}}^{{(2)}} \Gamma ^{T} P_{{\sigma (k_{{0,x}} )}} \Gamma  \\    \quad  + L^{T} D^{T} P_{{\sigma (k_{{0,x}} )}}^{T} Q_{{2,\sigma (k_{{0,x}} )}} P_{{\sigma (k_{{0,x}} )}} DL \\    \quad  - \Gamma ^{T} P_{{\sigma (k_{{0,x}} )}}^{T} Q_{{3,\sigma (k_{{0,x}} )}} P_{{\sigma (k_{{0,x}} )}} \Gamma  \\    \quad  + L^{T} D^{T} P_{{\sigma (k_{{0,x}} )}}^{T} Q_{{4,\sigma (k_{{0,x}} )}} P_{{\sigma (k_{{0,x}} )}} DL \\    \quad  - \Gamma ^{T} P_{{\sigma (k_{{0,x}} )}}^{T} Q_{{5,\sigma (k_{{0,x}} )}} P_{{\sigma (k_{{0,x}} )}} \Gamma  \\    \quad  - L^{T} NC_{{\sigma (k_{{0,x}} )}} D^{T} Q_{{6,\sigma (k_{{0,x}} )}}^{{ - 1}} DC_{{\sigma (k_{{0,x}} )}} L \\    \quad  - \Gamma ^{T} P_{{\sigma (k_{{0,x}} )}}^{T} Q_{{6,\sigma (k_{{0,x}} )}} P_{{\sigma (k_{{0,x}} )}} \Gamma ]x_{{ij}} (k - \tau )\}  \\     & \; = \left[ {\frac{{\lambda _{{\max }} (\Pi _{{\sigma (k_{{0,x}} )}} )}}{{\lambda _{{\min }} (P_{{\sigma (k_{{0,x}} )}}^{{ - 1}} )}} - 1} \right]V(k) + \frac{{\lambda _{{\max }} (\Omega _{{\sigma (k_{{0,x}} )}} )}}{{\lambda _{{\min }} (P_{{\sigma (k_{{0,x}} )}}^{{ - 1}} )}}V(k - \tau ) \\     & \; = \left[ {\frac{{\lambda _{{\max }} (\Pi _{{\sigma (k_{{0,x}} )}} )}}{{\lambda _{{\min }} (P_{{\sigma (k_{{0,x}} )}}^{{ - 1}} )}} + \mu q_{{\sigma (k_{{0,x}} )}} \frac{{\lambda _{{\max }} (\Omega _{{\sigma (k_{{0,x}} )}} )}}{{\lambda _{{\min }} (P_{{\sigma (k_{{0,x}} )}}^{{ - 1}} )}} - 1} \right]V(k) \\  \end{aligned}$$

Therefore, we obtain

37 $$\begin{aligned} V(\bar{k})&\leq\left[\frac{\lambda_{\rm max}(\Uppi_{\sigma(k_{0,x})})} {\lambda_{\rm min}(P_{\sigma(k_{0,x})}^{-1})} +\mu q_{\sigma(k_{0,x})}\frac{\lambda_{\rm max}(\Upomega_{\sigma(k_{0,x})})} {\lambda_{\rm min}(P_{\sigma(k_{0,x})}^{-1})}\right]V(\bar{k}-1) \\ & \quad \leq\left[\frac{\lambda_{\rm max}(\Uppi_{\sigma(k_{0,x})})} {\lambda_{\rm min}(P_{\sigma(k_{0,x})}^{-1})} +\mu q_{\sigma(k_{0,x})}\frac{\lambda_{\rm max}(\Upomega_{\sigma(k_{0,x})})} {\lambda_{\rm min}(P_{\sigma(k_{0,x})}^{-1})}\right]^{(\bar{k}-k^{\ast})}V(k^{\ast}) \\ & \quad \leq\left[\frac{\lambda_{\rm max}(\Uppi_{\sigma(k_{0,x})})} {\lambda_{\rm min}(P_{\sigma(k_{0,x})}^{-1})} +\mu q_{\sigma(k_{0,x})}\frac{\lambda_{\rm max}(\Upomega_{\sigma(k_{0,x})})} {\lambda_{\rm min}(P_{\sigma(k_{0,x})}^{-1})}\right]^{(k_{1,x}-k_{0,x})} \\ & \quad \cdot M\varphi(\|\phi(\theta)\|_{\tau}^{2})\text{e}^{-\lambda(k_{1,x}-k_{0,x})} \text{e}^{-\varepsilon_{\sigma(k_{0,x})}(k_{1,x}-k_{0,x})} \\ & \quad \leq M\varphi(\|\phi(\theta)\|_{\tau}^{2})\text{e}^{-\lambda(k_{1,x}-k_{0,x})} \\ & \quad <V(\bar{k}). \\ \end{aligned}$$

We have a contradiction here and (19) is true. Next comes that we suppose the claim (18) holds for $\hbox{m}= 1,2,\ldots ,\bar{m}$, such that

38 $$V(k)\leq M\varphi(\|\phi(\theta)\|_{\tau}^{2})\text{e}^{-\lambda(k_{m,x}-k_{0,x})}, k\in [k_{m-1,x},k_{m,x}-1].$$

Correspondingly, we should prove the Eq. (18) holds for $\hbox{m}=\bar{m}+1$,

39 $$V(k)\leq M\varphi(\|\phi(\theta)\|_{\tau}^{2})\text{e}^{-\lambda(k_{\bar{m}+1,x}-k_{0,x})}, k\in [k_{\bar{m},x},k_{\bar{m}+1,x}-1].$$

Note that from (13) and condition (ii), we have

40 $$\begin{aligned} V(k_{\bar{m},x})&=\sum\limits_{i=1}^{N-1}\sum\limits_{j=i+1}^{N}x_{ij}^{T}(k_{\bar{m},x}-1) (I+J_u({k_{\bar{m},x}}))^{T} \\ &\cdot P_{\sigma(k_{\bar{m},x})}(I+J_u({k_{\bar{m},x}}))x_{ij}(k_{\bar{m},x}-1) \\ &\leq \mu \lambda_{\rm max}^{2}(I+J_u({k_{\bar{m},x}})) \\ &\cdot \sum\limits_{i=1}^{N-1}\sum\limits_{j=i+1}^{N}x_{ij}^{T} (k_{\bar{m},x}-1)P_{\sigma(k_{\bar{m},x})}x_{ij}(k_{\bar{m},x}-1) \\ &=\mu \lambda_{\rm max}^{2}(I+J_u({k_{\bar{m},x}}))V(k_{\bar{m},x}-1) \\ &\leq \mu \lambda_{\rm max}^{2}(I+J_u({k_{\bar{m},x}})) M\varphi(\|\phi(\theta)\|_{\tau}^{2})\text{e}^{-\lambda(k_{\bar{m},x}-k_{0,x})} \\ &<\mu \lambda_{\rm max}^{2}(I+J_u({k_{\bar{m},x}})) M\varphi(\|\phi(\theta)\|_{\tau}^{2}) \\ &\cdot \text{e}^{-\lambda(k_{\bar{m}+1,x}-k_{0,x})}\text{e}^{\varepsilon_{\sigma(k_{\bar{m},x})} (k_{\bar{m}+1,x}-k_{\bar{m},x})} \\ &\leq M\varphi(\|\phi(\theta)\|_{\tau}^{2})\text{e}^{-\lambda(k_{\bar{m}+1,x}-k_{0,x})} \text{e}^{-\varepsilon_{\sigma(k_{\bar{m},x})}(k_{\bar{m}+1,x}-k_{\bar{m},x})} \\ \end{aligned}$$

If (39) is not true, then there exists a natural number $\bar{k}$ satisfying

41 $$\bar{k}=\text{min}\left\{k\in[k_{\bar{m},x},k_{\bar{m}+1,x}-1]:V(k)> M\varphi(\|\phi(\theta)\|_{\tau}^{2})\text{e}^{-\lambda(k_{\bar{m}+1,x}-k_{0,x})}\right\}.$$

Consequently, we have

42 $$\bar{k}>k_{\bar{m},x} \quad \text {and} \quad V(\bar{k})>V(k_{\bar{m},x}).$$

Define

43 $$\begin{aligned} k^{\ast}&=\text {max}\left\{k\in [k_{\bar{m},x},\bar{k}]: V(k)\leq \right.\\ &\left.M\varphi(\|\phi(\theta)\|_{\tau}^{2})\text{e}^{-\lambda(k_{\bar{m}+1,x}-k_{0,x})} \text{e}^{-\varepsilon_{\sigma(k_{\bar{m},x})}(k_{\bar{m}+1,x}-k_{\bar{m},x})}\right\}. \end{aligned}$$

Thus, there exists $k^{\ast}$ such that $k^{\ast}<\bar{k}$.

For any $k\in[k^{\ast}+1,\bar{k}]$, we have

44 $$V(k)>M\varphi(\|\phi(\theta)\|_{\tau}^{2}) \cdot \text{e}^{-\lambda(k_{\bar{m}+1,x}-k_{0,x})}\text{e}^{-\varepsilon_{\sigma(k_{\bar{m},x})} (k_{\bar{m}+1,x}-k_{\bar{m},x})},$$

and for any $k\in[k^{\ast},\bar{k}]$,

45 $$V(k^{\ast})\leq V(k) \leq V(\bar{k}).$$

Then, for any $k\in[k_{\bar{m},x},k_{\bar{m}+1,x}-1]$, we can obtain

46 $$V(k+1)\leq \left[\frac{\lambda_{\rm max}(\Uppi_{\sigma(k_{\bar{m},x})})} {\lambda_{\rm min}(P_{\sigma(k_{\bar{m},x})}^{-1})}+\mu q_{\sigma(k_{\bar{m},x})} \frac{\lambda_{\rm max}(\Upomega_{\sigma(k_{\bar{m},x})})} {\lambda_{\rm min}(P_{\sigma(k_{\bar{m}-m_{\tau},x})}^{-1})}\right]V(k).$$

According to the condition (iii), one observes that for any ${s\in \mathbb{Z}_{\tau},\;k+s\in [k_{\bar{m}-m_{\tau},x},\bar{k}]}$.

Meanwhile for any $k\in[k^{\ast},\bar{k}-1]$,

47 $$\begin{aligned} V(k+s)&\leq M\varphi(\|\phi(\theta)\|_{\tau}^{2})\text{e}^{-\lambda(k_{\bar{m}-m_{\tau}+1,x}-k_{0,x})} \\ &\leq M\varphi(\|\phi(\theta)\|_{\tau}^{2})\text{e}^{-\lambda(k_{\bar{m}+1,x}-k_{0,x})} \\ &\cdot \text{e}^{\sum\nolimits_{i=0}^{m_{\tau}-1}\varepsilon_{\sigma(k_{\bar{m}-i,x})} (k_{\bar{m}+1-i,x}-k_{\bar{m}-i,x})} \\ &\leq \text{e}^{-\varepsilon_{\sigma(k_{\bar{m},x})}(k_{\bar{m}+1,x}-k_{\bar{m},x})} M\varphi(\|\phi(\theta)\|_{\tau}^{2})\text{e}^{-\lambda(k_{\bar{m}+1,x}-k_{0,x})} \\ &\cdot \text{e}^{\varepsilon_{\sigma(k_{\bar{m},x})}(k_{\bar{m}+1,x}-k_{\bar{m},x})} \text{e}^{\sum\nolimits_{i=0}^{m_{\tau}-1}\varepsilon_{\sigma(k_{\bar{m}-i,x})} (k_{\bar{m}+1-i,x}-k_{\bar{m}-i,x})} \\ &\leq \text{e}^{\varepsilon_{\sigma(k_{\bar{m},x})}(k_{\bar{m}+1,x}-k_{\bar{m},x}) +\sum\limits_{i=0}^{m_{\tau}-1}\varepsilon_{\sigma(k_{\bar{m}-i,x})} (k_{\bar{m}+1-i,x}-k_{\bar{m}-i,x})} \\ & \cdot V(k^{\ast}+1) \\ &< \text{e}^{\varepsilon_{\sigma(k_{\bar{m},x})}(k_{\bar{m}+1,x}-k_{\bar{m},x}+1) +\sum\limits_{i=0}^{m_{\tau}-1} \varepsilon_{\sigma(k_{\bar{m}-i,x})}(k_{\bar{m}+1-i,x}-k_{\bar{m}-i,x})} \\ & \cdot V(k^{\ast}) \\ &\leq q_{\sigma(k_{\bar{m},x})}V(k). \\ \end{aligned}$$

Consider that $k=\bar{k}$, we have

48 $$\begin{aligned} V(\bar{k})&\leq \left[\frac{\lambda_{\rm max}(\Uppi_{\sigma(k_{\bar{m},x})})} {\lambda_{\rm min}(P_{\sigma(k_{\bar{m},x})}^{-1})} \right.\\ &\left.+\mu q_{\sigma(k_{\bar{m},x})}\frac{\lambda_{\rm max}(\Upomega_{\sigma(k_{\bar{m},x})})} {\lambda_{\rm min}(P_{\sigma(k_{\bar{m}-m_{\tau},x})}^{-1})}\right]^{(\bar{k}-k^{\ast})} V(k^{\ast}) \\ &\leq \left[\frac{\lambda_{\rm max}(\Uppi_{\sigma(k_{\bar{m},x})})} {\lambda_{\rm min}(P_{\sigma(k_{\bar{m},x})}^{-1})} \right.\\ &\left.+\mu q_{\sigma(k_{\bar{m},x})}\frac{\lambda_{\rm max}(\Upomega_{\sigma(k_{\bar{m},x})})} {\lambda_{\rm min}(P_{\sigma(k_{\bar{m}-m_{\tau},x})}^{-1})}\right] ^{(k_{\bar{m}+1,x}-k_{\bar{m},x})} \\ &\cdot M\varphi(\|\phi(\theta)\|_{\tau}^{2})\text{e}^{-\lambda(k_{\bar{m}+1,x}-k_{0,x})} \text{e}^{-\varepsilon_{\sigma(k_{\bar{m},x})}(k_{\bar{m}+1,x}-k_{\bar{m},x})} \\ &<M\varphi(\|\phi(\theta)\|_{\tau}^{2})\text{e}^{-\lambda(k_{\bar{m}+1,x}-k_{0,x})} \\ &<V(\bar{k}), \\ \end{aligned}$$

which is a contradiction. Hence, the Eq. (39) holds for $k=\bar{k}+1$. And by virtue of mathematical induction, the claim (18) is true for each ${k\in \mathbb{N}}$.

In view of (18) and **Definition 1**, it can be obtained that

49 $$V(k)\leq M\varphi(\|\phi(\theta)\|_{\tau}^{2})\text{e}^{-\lambda(k-k_{0,x})}, k\in[k_{m-1,x},k_{m,x}-1], m\in{\mathbb{N}}.$$

For any $${k\in\mathbb{N}}$$,

50 $$\begin{aligned} &\text{min}\left\{\lambda_{\rm min}(P_{\sigma(k)})\right\} \sum\limits_{i=1}^{N-1}\sum\limits_{j=i+1}^{N}\|x_i(k)-x_j(k)\|^{2} \\ &\;\leq \sum\limits_{i=1}^{N-1}\sum\limits_{j=i+1}^{N}(x_i(k)-x_j(k))^{T}P_{\sigma(k)} (x_i(k)-x_j(k)) \\ &\;=V(k) \\ &\;\leq M\varphi(\|\phi(\theta)\|_{\tau}^{2})\text{e}^{-\lambda(k-k_{0,x})}. \\ \end{aligned}$$

Therefor, for any ${k\in \mathbb{N}}$,

51 $$\begin{aligned} &\sum\limits_{i=1}^{N-1}\sum\limits_{j=i+1}^{N}\|x_i(k)-x_j(k)\|^{2} \\ &\;\leq \text{min}\left\{\lambda_{\rm min}^{-1}(P_{\sigma(k)})\right\} M\varphi(\|\phi(\theta)\|_{\tau}^{2})\text{e}^{-\lambda(k-k_{0,x})}, \\ \end{aligned}$$

which implies

52 $$\|x_i(k)-x_j(k)\|\leq M_0\text{e}^{-\lambda(k-k_{0,x})},\quad 1\leq i \leq j\leq N.$$

Therefore, the discrete complex networks (1) are globally exponentially synchronized under impulsive control. The proof is thus completed. □

**Remark**. We consider a multiple Lyapunov function for each sub-network with arbitrarily fast switching signal in our theorem, which results in a less conservation criterion.

### *Remark 1*

In the switched Lyapunov function, *p*_σ(*k*)_ gives an upper bound on the estimation of divergence rate for each running sub-network. By condition (ii) of **Theorem 1**, the impulsive control gain is designed to compensate divergence from system itself and deteriorating effect from arbitrarily fast switching. If some certain sub-networks could be self-synchronizing, the impulsive control gain only needs to compensate deteriorating effect.

## Example and numerical simulations

This section presents a typical example to illustrate our result. Let us consider a 2-dimensional discrete chaotic neural network is given as the isolated node of a small-world network with 30 nodes,

53 $$x(k+1)=Ax(k)+Bf(x(k))+Df(x(k-\tau(k)))+I(k),$$

where $$x(k)=(x_1(k),x_2(k))^{T},\;f(x(k))=(\text{tan}h(x_1(k)),\;\text{tan}h(x_2(k)))^{T},\;I(k)=(0,0)^T, \;A=\left[\begin{array}{cc} -1 & 0 \\ 0 & -1\end{array}\right],\;B=\left[\begin{array}{cc} 2 & -0.11 \\ -5 & 3.2\end{array}\right],\;D=\left[\begin{array}{cc} -1.6 & -0.1 \\ -0.18 & -2.4\end{array}\right],$$ and $\tau(k)=\frac{\text{e}^{k}}{1+\text{e}^{(k)}}.$ Obviously, Lipschitz constants can be 1 here. Consider a small-world model involved with three different sub-systems as follow

**Fig. 1:**
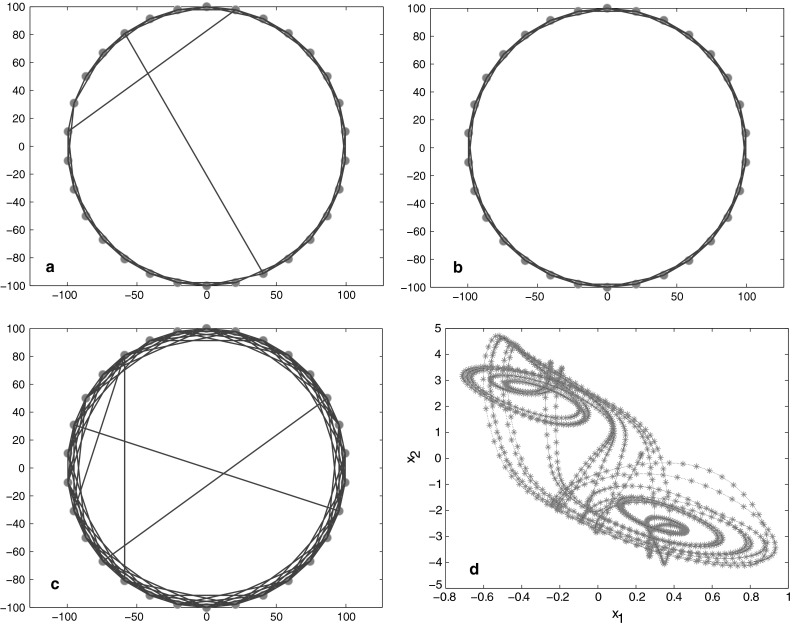
**a** *N* = 30, *k* = 2, *p* = 0.02; **b** *N* = 30, *k* = 2, *p* = 0.01; **c** *N* = 30, *k* = 4, *p* = 0.04; **d** Chaotic trajectory of each single node

- 30 nodes are arranged in a ring, while each node *i* is adjacent to its neighbor node; each pair of nodes are coupled to the whole network by probability *p* = 0.02. See Fig. 1a.
- 30 nodes are arranged in a ring, while each node *i* is adjacent to its neighbor node; each pair of nodes are coupled to the whole network by probability *p* = 0.01. See Fig. 1b.
- 30 nodes are arranged in a ring, while each node *i* is adjacent to its 2 neighbor node; each pair of nodes are coupled to the whole network by probability *p* = 0.04. See Fig. 1c.

The trajectory of each single node of this small-world model has been portrayed in Fig. 1d with random initial values in the interval [0.3, 3] and [−3, −0.3], respectively.

Let $\Upgamma=diag\{0.15,0.3\}$, we can obtain the state response of each sub-network. Note that there is no switched rule in each sub-network here, and the initial values are randomly chosen in the interval [0.3, 3] and [−3, −0.3], respectively.

Given a switching signal σ(*t*) in Fig. 3a, we have the state response of the switched complex networks, see Fig. 3b. From **Theorem 1**, for each sub-network, we have

- $$P_{1}=\left[\begin{array}{cc} 3.0001 & 0 \\ 0 & 3.0001\end{array}\right],\;Q_{1,1}=\left[\begin{array}{cc} 5.0690 & 0 \\ 0 & 5.0690\end{array}\right],\;Q_{2,1}=\left[\begin{array}{cc} 9.3812 & 0 \\ 0 & 9.3812\end{array}\right],\;Q_{3,1}=\left[\begin{array}{cc} 33.7971 & 0 \\ 0 & 33.7971\end{array}\right],\;Q_{4,1}=\left[\begin{array}{cc} 11.4594 & 0 \\ 0 & 11.4594\end{array}\right],\;Q_{5,1}=\left[\begin{array}{cc} 50.9300 & 0 \\ 0 & 50.9300\end{array}\right],\;Q_{6,1}=\left[\begin{array}{cc} 20.1879 & 0 \\ 0 & 20.1879\end{array}\right]$$. And $$\varepsilon_1=0.6213,\;q_1<22.42$$. Thus, $$J_{1}=\left[\begin{array}{cc} -0.6667 & 0 \\ 0 & -0.667\end{array}\right]$$.
- $$P_{2}=\left[\begin{array}{cc} 2.9998 & 0 \\ 0 & 2.9998\end{array}\right],\;Q_{1,2}=\left[\begin{array}{cc} 5.1333 & 0 \\ 0 & 5.1333\end{array}\right],\;Q_{2,2}=\left[\begin{array}{cc} 9.9398 & 0 \\ 0 & 9.9398\end{array}\right],\;Q_{3,2}=\left[\begin{array}{cc} 29.7933 & 0 \\ 0 & 29.7933\end{array}\right],\;Q_{4,2}=\left[\begin{array}{cc} 11.2764 & 0 \\ 0 & 11.2764\end{array}\right],\;Q_{5,2}=\left[\begin{array}{cc} 52.0308 & 0 \\ 0 & 52.0308\end{array}\right],\;Q_{6,2}=\left[\begin{array}{cc} 19.9970 & 0 \\ 0 & 19.9970\end{array}\right]$$. And $$\varepsilon_2=0.8870,\;q_2<4.22$$. Thus, $$J_{2}=\left[\begin{array}{cc} -0.4079 & 0 \\ 0 & -0.4079\end{array}\right]$$.
- $$P_{3}=\left[\begin{array}{cc} 13.4267 & 0 \\ 0 & 13.4267\end{array}\right],\;Q_{1,3}=\left[\begin{array}{cc} 10.2609 & 0 \\ 0 & 10.2609\end{array}\right],\;Q_{2,3}=\left[\begin{array}{cc} 3.1001 & 0 \\ 0 & 3.1001\end{array}\right],\;Q_{3,3}=\left[\begin{array}{cc} 16.6667 & 0 \\ 0 & 16.6667\end{array}\right],\;Q_{4,3}=\left[\begin{array}{cc} 5.2060 & 0 \\ 0 & 5.2060\end{array}\right],\;Q_{5,3}=\left[\begin{array}{cc} 30.8123 & 0 \\ 0 & 30.8123\end{array}\right],\;Q_{6,3}=\left[\begin{array}{cc} 3.0112 & 0 \\ 0 & 3.0112\end{array}\right]$$. And $$\varepsilon_3=0.1062,\;q_3<12.62$$. Thus, $$J_{3}=\left[\begin{array}{cc} -1.1576 & 0 \\ 0 & -1.1576\end{array}\right]$$.

**Fig. 2:**
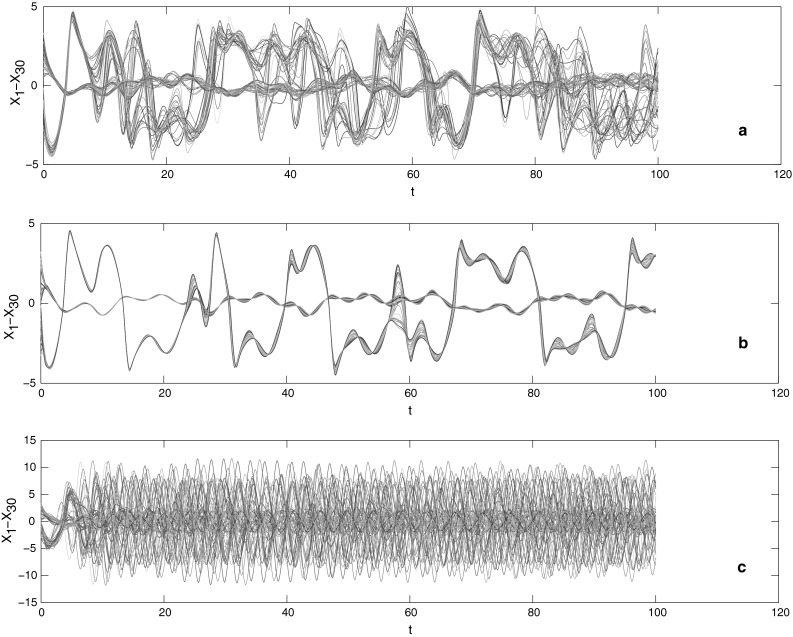
**a** The state responses of sub-networks 1; **b** the state responses of sub-networks 2; **c** the state responses of sub-networks 3

**Fig. 3:**
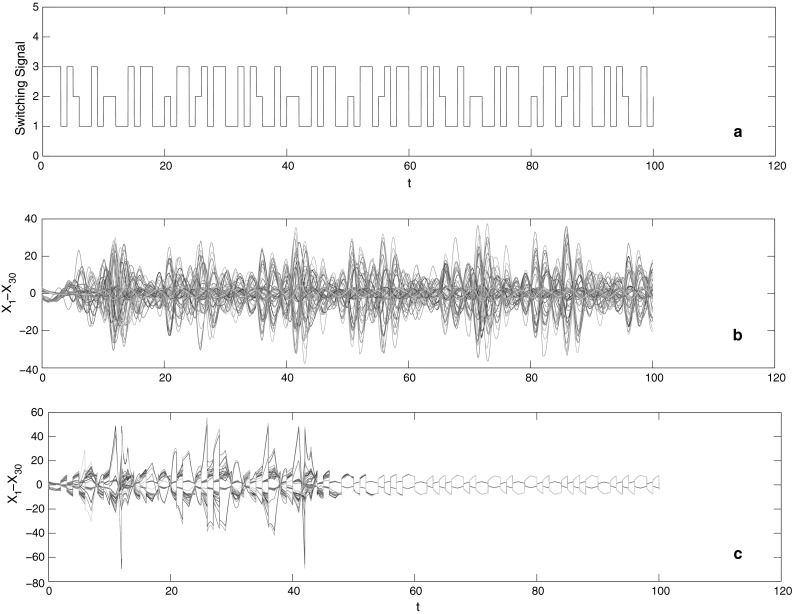
**a** The switching signal σ(t); **b** the state responses of the switched system; **c** the synchronized state under impulsive control

It is shown from Fig. 2a–c that all of nodes in each sub-network could not reach into a synchronous state without a control. Indeed, the switched signal plays a role of deterioration accelerator to diverge the synchronous state, shown in Fig. 3b. Once the feasible impulsive controller is placed on discrete complex networks with topology switching, such complex networks would be synchronized, see Fig. 3c.

## Conclusion

In this paper, we have investigated impulsive synchronization control of a discrete delayed complex network with switching topology by using Lyapunov-Ruzimiki method. A time-varying delay-dependent criteria for exponential synchronization is presented guarantee the switched discrete complex networks tending to be a synchronous manifold. It is worthwhile to see time-varying delay can take any value, even larger than any dwell time of a sub-network. Furthermore, a numerical example with three sub-networks is presented by using the impulsive control technique.
